# Ivabradine in treatment of symptomatic heart failure and supraventricular tachycardias in patients under six months of age

**DOI:** 10.3389/fphar.2025.1502375

**Published:** 2025-02-18

**Authors:** L. Gómez-Ganda, C. J. Parramón-Teixidó, G. Giralt-García, C. Fernández-García, Q. Ferrer-Menduiña, M. J. Cabañas-Poy

**Affiliations:** ^1^ Pharmacy Department, Vall d’Hebron University Hospital, Barcelona, Spain; ^2^ Pediatric Cardiology Department, Vall d’Hebron University Hospital, Barcelona, Spain; ^3^ Neonatology Department, Vall d’Hebron University Hospital, Barcelona, Spain

**Keywords:** heart failure, ivabradine, pediatrics, tachycardia, atrial tachycardia, ectopic atrial tachycardia, supraventricular tachycardia, junctional ectopic tachycardia

## Abstract

**Background:**

Although heart failure (HF) and supraventricular tachycardias (SVT) are associated with high morbidity and mortality in pediatrics, especially, in children under 6 months; the efficacy of available treatments is limited, requiring the use of off-label therapies. The aim of the study is to investigate the efficacy, dosage, and safety of off-label ivabradine in patients under 6 months of age with HF or SVT.

**Methods:**

Retrospective observational study, which included patients under 6 months of age with HF or SVT who received ivabradine between January 2020 - May 2024. Demographic, clinical, and treatment-related variables were collected. Response variables were established according to indication, HF: heart rate (HR) and left ventricular ejection fraction (LVEF); SVT: HR.

**Results:**

Thirteen patients (nine women) with a median age of 1.4 (1-4) months were included. Ivabradine was discontinued in five of the seven HF patients due to resolution of HF, control of HR, and improvement of LVEF. One patient discontinued ivabradine because of bradycardia. In the SVT group, four of the seven patients discontinued ivabradine after the resolution of tachyarrhythmia and improvement of HR. Two patients experienced bradycardia but did not require treatment discontinuation. HR reduction was statistically significant in both groups. In HF, the median initial ivabradine dose was 0.06 mg/kg/day and the maintenance dose was 0.2 mg/kg/day. In SVT, the initial and maintenance doses were 0.1 mg/kg/day and 0.24 mg/kg/day, respectively.

**Conclusion:**

Ivabradine demonstrated favorable efficacy and safety results in patients under 6 months of age with HF or SVT.

## Highlights

• First published study that reports the use of ivabradine in patients under 6 months of age with heart failure.
• Study with the largest cohort of patients under 6 months who have received ivabradine for a diagnosis of supraventricular tachycardia.
• Ivabradine improved clinical parameters with an appropriate safety profile in patients under 6 months with heart failure and supraventricular tachycardia, presenting an effective and safe therapeutic alternative.
• Reduction in heart rate in patients with heart failure and supraventricular tachycardia was statistically significant.

## 1 Introduction

Heart failure (HF) and cardiac arrhythmias are significant causes of morbidity and mortality in the pediatric population, particularly during the perinatal period (Bonnet et al., 2017; Marín-García, 2004).

### 1.1 Heart failure (HF)

Congenital heart diseases (CHD) represent the leading cause of HF in the pediatric population (Gómez-Guzmán et al., 2013). Approximately 90% of patients with CHD develop HF within the first year of life, with the highest incidence occurring during the first 6 months (SECPCC, 2015). The second most common cause of HF are primary cardiomyopathies, particularly dilated cardiomyopathy (DCM), which has a higher incidence and worse prognosis in patients under 1 year of age (Bonnet et al., 2017; Gómez-Guzmán et al., 2013).

Currently, the treatment of HF aims to alleviate symptoms, slow disease progression, and reduce associated mortality. To achieve this, therapies that improve cardiac function and decrease heart rate (HR) are necessary, as elevated HR values are sometimes associated with an increased risk of death (Bonnet et al., 2017). However, the heterogeneity of the condition and its low incidence make standardizing treatment in pediatrics challenging (Gómez-Guzmán et al., 2013). Guidelines recommend the use of diuretics, digoxin, angiotensin-converting enzyme inhibitors (ACEIs), aldosterone antagonists, and beta-blockers (Bonnet et al., 2017; SECPCC, 2015). Nonetheless, due to the lack of evidence and controlled clinical trials in pediatric patients, treatment recommendations are often extrapolated from adult data. Consequently, most drugs are not approved for pediatric use (Bonnet et al., 2017).

### 1.2 Supraventricular tachycardias (SVT)

Tachyarrhythmias, a subset of cardiac arrhythmias, are characterized by heart rates exceeding the normal limits for age, with supraventricular tachycardias (SVT) being the most common in pediatrics (SECPCC, 2015). The age distribution of pediatric SVT is bimodal, with peaks occurring in infants under 1 year and in children between seven and 12 years of age (SECPCC, 2015). SVT typically present in patients without structural heart disease, although it can also be associated with CHD or cardiomyopathies, as well as secondary to surgical interventions (SECPCC, 2015).

SVT include conditions such as junctional ectopic tachycardia (JET), focal atrial tachycardia (FAT), multifocal atrial tachycardia (MAT), and ectopic atrial tachycardia (EAT) (SECPCC, 2015).

The increase in HR is associated with greater myocardial oxygen demand and impaired diastolic perfusion. Therefore, the treatment aims to achieve adequate HR control to meet myocardial oxygen demands and prevent potential complications (Younis et al., 2021). This treatment involves the administration of antiarrhythmic agents such as amiodarone, digoxin, and flecainide; however, a lack of response and resistance to these therapies is commonly observed (Younis et al., 2021; Michel et al., 2020).

### 1.3 Ivabradine in the pediatric population

Given the lack of effective therapeutic alternatives for treating HF, as well as the frequent resistance to conventional therapies in the case of SVT in pediatric patients, the use of oral ivabradine has been proposed for both conditions.

Currently, ivabradine is approved by the European Medicines Agency (EMA) as an antiarrhythmic agent exclusively for adult patients (EMA, 2024). However, while the U.S. Food and Drug Administration (FDA) initially approved ivabradine for use only in adult patients, it later extended its indication to pediatric patients for the treatment of stable symptomatic HF due to DCM from 6 months of age (Bonnet et al., 2017; FDA, 2024).

Ivabradine has also been used off-label for HR control in pediatric patients with various SVT, such as JET, FAT, MAT, and EAT, demonstrating efficacy and good tolerability (Younis et al., 2021; Kumar et al., 2019; Kumar et al., 2017; Tolani et al., 2024; Dieks et al., 2016). However, the experience with ivabradine in these indications is limited and primarily based on case reports or case series, particularly in patients under 6 months of age, for whom no dosing recommendations have been established.

Thus, data on the efficacy, safety, and dosing of ivabradine in patients younger than 6 months remain very limited. Nevertheless, HF caused by CHD or DCM, as well as SVT, have a higher incidence in this population. This highlights an urgent need for further research on its use in this age group.

The present study aims to evaluate the efficacy and safety of off-label ivabradine use in patients under 6 months of age and to describe the dosing regimens employed.

## 2 Materials and methods

### 2.1 Study design and patient selection

Retrospective observational study conducted at a national referral center for both pediatric cardiology and neonatology. The study included all pediatric patients who initiated ivabradine treatment before 6 months of age between January 2020 and May 2024.

Prior to starting ivabradine therapy, as per the hospital’s standard practice, parents or legal guardians were informed about the off-label use of the drug and its potential side effects.

### 2.2 Data collection

The study was reviewed and approved by the center’s Research Ethics Committee for medicinal products [EOM(AMI)054/2024 (6325)]. After obtaining approval, patients who had received ivabradine treatment within the first 6 months of life during the study period were identified and selected through the electronic prescribing system. The following parameters were collected in an anonymized database (Microsoft Excel^®^ 2016) from the electronic medical records: demographic and clinical variables, indication for ivabradine treatment, variables related to ivabradine use, clinical parameters at the start and end of treatment, and adverse effects.

Patients were classified into two groups based on the indication for ivabradine to evaluate treatment response according to associated clinical variables: i) symptomatic HF: HR and left ventricular ejection fraction (LVEF), and ii) SVT: HR. LVEF was determined via echocardiography using the Teichholz and Simpson methods. The final treatment values of these variables correspond to those recorded at the time of discontinuation or, for patients still receiving therapy, to the latest values measured before the study’s conclusion.

### 2.3 Compounded formulation of ivabradine

Since ivabradine in Europe is only approved for adult patients, it is commercially available only in tablet form at doses defined for this population, complicating its administration in pediatric patients, especially in those under 6 months of age who require lower doses. Although ivabradine is not classified as a hazardous drug by the U.S. National Institute for Occupational Safety and Health (NIOSH), it could be considered hazardous due to its teratogenic potential, and safety precautions are recommended during its handling (National Institute for Occupational Safety and Health NIOSH, 2020).

To ensure accurate dosing and increase safety during handling, the Pharmacy Department decided to prepare individualized ivabradine capsules for each patient, starting from the commercially available tablet form, using lactose as the excipient.

### 2.4 Statistical analysis of results

A descriptive statistical analysis was conducted for the demographic and clinical variables of the patients. The results are presented as mean (standard deviation), median (interquartile range), absolute frequencies, and percentages, depending on the nature and distribution of the data.

To evaluate treatment response, the reduction in HR was calculated in both groups, and in the symptomatic HF group, the change in LVEF was also determined. Normality of the variables was assessed graphically and through Skewness/Kurtosis and Shapiro-Wilk tests. If the normality assumption was met, the paired Student’s t-test was used; otherwise, the Wilcoxon signed-rank test was applied. Statistical analysis was performed using StataCorp. 2019 (Stata Statistical Software: Release 16. College Station, TX: StataCorp LLC).

## 3 Results

During the study period, 13 patients under 6 months of age (nine women) initiated treatment with ivabradine, with a median age of 1.4 (1 - 4) months. Table 1 details the patients diagnosed with symptomatic HF, while Table 2 presents those diagnosed with SVT. Patient 7 is listed in both groups, as she initially received ivabradine for sinus tachycardia and, after completing that treatment, was later diagnosed with severe diastolic dysfunction and resumed ivabradine therapy. Individualized ivabradine capsules were prepared by the Pharmacy Department for all patients included in the study.

**TABLE 1:** Patients who received treatment with ivabradine for a diagnosis of symptomatic HF.

Demographic and clinical variables							
	Patient 1	Patient 2	Patient 3	Patient 4	Patient 5	Patient 6	Patient 7
Sex	W	W	W	M	M	W	W
Gestational age at birth (GW)	39 + 5	39 + 1	26 + 4	38 + 1	38 + 2	39 + 4	37 + 1
Birth weight (kg)	2.7	3.3	1.1	4.1	2.7	3.3	2.7
Medical history	Sengers syndrome (mitochondrial disease)	-	MYH7 genetic disorder	-	Propionic acidemia	-	Trisomy 21, AV canal heart defect
Baseline variables at the initiation of ivabradine treatment							
Indication	Congenital hypertrophic cardiomyopathy with moderate LV dysfunction and mild RV dysfunction	Left-sided hypoplasia type III with mild RV dysfunction and severe systemic insufficiency	Genetic origin DCM with severe biventricular dysfunction	Left-sided hypoplasia with mitroaortic atresia, palliated with the Norwood procedure and central shunt	Left-sided DCM with severe dysfunction	Congenital cardiomyopathy with moderate LV dysfunction and mild RV dysfunction	Severe diastolic dysfunction
Age (months)	4.4	2.5	4	30 days	3.6	7 days	6.2
Weight (kg)	5.4	4.0	3.5	4.2	3.7	3.1	6.5
Time between diagnosis and initiation of treatment (days)	124	70	111	28	0	7	0
Dose (mg/kg/day every 12 h)	0.1	0.1	0.1	0.2	0.05	0.06	0.06
Route of administration	NG tube	NG tube	NG tube	NG tube	NG tube	OR	NG tube
Concomitant treatments	Captopril, carvedilol	Milrinone, levosimendan, furosemide, digoxin	Captopril, furosemide, carvedilol	Milrinone, furosemide, digoxin	Furosemide	Digoxin	Captopril, furosemide, spironolactone
HR (bpm)	150	130	160	160	150	160	140
LVEF (%) Teichholz/Simpson	45/50	40/NA	25/22	NA/64	30/30	45/48	79/NA
Variables at the end of ivabradine treatment or at the time of study closure							
Discontinuation of treatment	Yes	Yes	Yes	Yes	Yes	Yes	Yes
Reason for discontinuation of treatment	Postoperative death following cataract surgery	Resolution (subject to various surgical interventions)	Bradycardia	Resolution	Resolution	Resolution	Resolution
Duration of treatment	9 days	18.5 months	13 months	8.7 months	4.5 months	15 days	3.7 months
Dose (mg/kg/day every 12 h)	0.18	0.2	0.2	0.2	0.2	0.1	0.1
Concomitant treatments	Captopril, carvedilol	Captopril, furosemide, digoxin	Furosemide, hydrochlorothiazide, digoxin	Captopril, furosemide, digoxin	Captopril, furosemide, spironolactone, carvedilol	Digoxin	No
HR (bpm)	100	125	50 - 60	115	120	150	120
LVEF (%) Teichholz/Simpson	NA/NA	40/NA	36/33	NA/65	52/52	47/43	83/NA
Related adverse effects	No	No	Gastrointestinal, bradycardia	No	QT interval prolongation	Bradycardia	No

AV: atrioventricular; bpm: beats per minute; DCM: dilated cardiomyopathy; GW: gestational weeks; HF: heart failure; HR: heart rate; LV: left ventricular; LVEF: left ventricular ejection fraction; M: man; NA: not available; NG: nasogastric; OR: oral; RV: right ventricular; W: woman.

**TABLE 2:** Patients who received ivabradine treatment for a diagnosis of SVT.

Demographic and clinical variables								
	Patient 8		Patient 9	Patient 10	Patient 11	Patient 12	Patient 13	Patient 7
Sex	M		W	W	W	W	M	W
Gestational age at birth (GW)	41 + 5		37 + 1	40 + 0	37 + 3	38 + 2	40 + 3	37 + 1
Birth weight (kg)	NA		2.3	3.7	3.0	3.2	ND	2.7
Medical history	-		-	-	Transposition of the great vessels	-	-	Trisomy 21, AV canal heart defect
Baseline variables at the initiation of ivabradine treatment								
Indication	Congenital JET		FAT	FAT	Postoperative anomalous sinus tachycardia	Myocardial ischemia with elevated HR	FAT	Postoperative JET
Age (months)	5.1		20 days	30 days	9 days	1.2	1.4	5.7
Weight (kg)	7.0		2.4	4	3010	3.4	4.5	6.9
Time between diagnosis and initiation of treatment (days)	5		3	3	1	11	37	2
Dose (mg/kg/day every 12 h)	0.1		0.1	0.1	0.07	0.1	0.1	0.1
Route of administration	OR		NG tube	NG tube	NG tube	NG tube	NG tube	OR
Concomitant treatments	Propranolol		Furosemide, propranolol, flecainide	Amiodarone	Milrinone, furosemide, dopamine	Propranolol	Furosemide, esmolol, flecainide	Milrinone, amiodarone, furosemide, spironolactone
HR (bpm)	190		140	175	200	150	180	150 - 160
Variables at the end of ivabradine treatment or at the time of study closure								
Discontinuation of treatment		No	No	No	Yes	Yes	Yes	Yes
Reason for discontinuation of treatment		-	-	-	Resolution	Resolution	Resolution	Resolution
Duration of treatment		36 months	18.1 months	82 days	1 dose	18.6 months	56 days	1 day
Dose (mg/kg/day every 12 h)		0.4	0.24	0.1	0.07	0.3	0.34	0.1
Concomitant treatments		Propranolol	Flecainide	Propranolol	Milrinone, furosemide	Captopril, propranolol	Propranolol, flecainide	Furosemide, spironolactone
HR (bpm)		80	125	125	125	103	119	135 - 145
Related adverse effects		No	Bradycardia	No	No	Self-limited bradycardia	No	No

AV: atrioventricular; bpm: beats per minute; FAT: focal atrial tachycardia; GW: gestational weeks; HR: heart rate; JET: junctional ectopic tachycardia; M: man; NA: not available; NG: nasogastric; OR: oral; SVT: supraventricular tachycardia; W: woman.

Table 3 presents the descriptive statistics and statistical analysis of the demographic and clinical variables of patients classified by diagnosis, as well as the results before and after ivabradine treatment.

**TABLE 3:** Descriptive statistics and analysis of demographic and clinical variables of patients by diagnosis.

			Symptomatic HF n = 7	SVT n = 7
Demographic and clinical variables				
Women		n (%)	5 (71)	5 (71)
Gestational age at birth (GW)		Mean (SD)	36.5 (4.5)	38.6 (1.6)
Birth weight (kg)		Median (range)	2.99 (2.7 - 3.33)	3.11 (2.67 - 3.2)
Baseline variables at the initiation of ivabradine treatment				
Age (months)		Median (range)	3.6 (1.75 - 4.2)	1.2 (0.9 - 3.25)
Weight (kg)		Median (range)	3.95 (3.62 - 4.82)	4 (3.2 - 5.5)
Time between diagnosis and initiation of treatment (days)		Median (range)	28 (3.5 - 89.5)	3 (2.5 - 8)
Dose (mg/kg/day every 12 h)		Median (range)	0.06 (0.057 - 0.1)	0.1 (0.1 - 0.12)
Route of administration	OR	n (%)	1 (14)	2 (29)
	NG tube	n (%)	6 (86)	5 (71)
Number of concomitant treatments		Median (range)	3 (1.5 - 3)	2 (1 - 3)
HR (bpm)		Mean (SD)	152.1 (13.5)	170.7 (21.7)
LVEF (%)	Teichholz	Mean (SD)	44 (19)	-
	Simpson	Mean (SD)	42.9 (16.8)	-
Variables at the end of ivabradine treatment or at the time of study closure				
Discontinuation of treatment		n (%)	7 (100)	4 (57)
Duration of treatment		Median (range)	4.5 (2.1 - 10.85)	2.7 (0.85 - 18.35)
Dose (mg/kg/day every 12 h)		Median (range)	0.2 (0.14 - 0.2)	0.24 (0.12 - 0.32)
Number of concomitant treatments		Median (range)	3 (1.5 - 3)	2 (1 - 2)
HR (bpm)		Mean (SD)	111.4 (30.9)	116 (18.6)
Reduction in HR (bpm)		Mean (SD) P value	−40.7 (36.0) p = 0.04	−54.7 (31.8) p = 0.02
LVEF (%)	Teichholz	Mean (SD)	51.6 (18.6)	-
	Simpson	Mean (SD)	48.3 (13.6)	-
Increment in LVEF (%)	Teichholz	Mean (SD); p value	7.8 (9); p = 0.12	-
	Simpson	Mean (SD); p value	7.3 (11.8); p = 0.38	-

bpm: beats per minute; GW: gestational weeks; HF: heart failure; HR: heart rate; LVEF: left ventricular ejection fraction; NG: nasogastric; OR: oral; SD: standard deviation; SVT: supraventricular tachycardia.

None of the patients in the symptomatic HF group continued on ivabradine therapy. In five of the seven patients (71.4%), treatment was discontinued following resolution of dysfunction, with adequate HR control observed in all of them (mean reduction of 41 beats per minute (bpm)) and an improvement in LVEF (mean increase of 7%–8%). Treatment was only discontinued in one patient due to adverse effects (bradycardia). For patients with SVT, treatment was discontinued in four of the seven patients (57.1%) following resolution of the tachyarrhythmia and improvement in HR (mean reduction of 55 bpm), and no discontinuation was required due to adverse effects, although two patients experienced bradycardia during treatment, with one case being self-limiting. In both groups, the reduction in HR was statistically significant.

Regarding the dosing of ivabradine for symptomatic HF, the median starting dose was 0.06 (0.057 - 0.1) mg/kg/day and the maintenance dose was 0.2 (0.14 - 0.2) mg/kg/day. In the case of SVT, the doses were 0.1 (0.1 - 0.12) mg/kg/day for the starting dose and 0.24 (0.12 - 0.32) mg/kg/day for the maintenance dose. The median duration of treatment for patients with symptomatic HF was 4.5 (2.1 - 10.9) months, while for patients with SVT, it was 2.7 (0.9 - 18.35) months.

## 4 Discussion

Certain cardiac conditions, such as HF and SVT, are associated with high morbidity and mortality in the pediatric population, particularly in patients under 6 months and 1 year of age (Bonnet et al., 2017; Marín-García, 2004). Despite the severity of these conditions, treatment evidence is limited due to a lack of studies in pediatrics. This results in restricted pharmacological options and, at times, inadequate disease management due to poor response or resistance to conventional treatments. Consequently, there is a need for the use of off-label therapies and/or those with limited evidence, such as ivabradine.

The mechanism of action of ivabradine is based on the selective and specific inhibition of the cardiac pacemaker current (If), which controls spontaneous diastolic depolarization in the sinus node and regulates HR; this results in a dose-dependent reduction in HR and myocardial oxygen consumption (EMA, 2024). Because its cardiac effects are specific to the sinus node, ivabradine exhibits negative inotropic activity, which may present a safer alternative in patients with decompensated systolic HF, compared to beta-blockers (Younis et al., 2021; EMA, 2024). Adverse effects are dose-dependent and arise from its mechanism of action, with frequent side effects including luminous phenomena (phosphenes), bradycardia, atrial fibrillation, headaches, dizziness, blurred vision, and hypotension. Gastrointestinal adverse effects and QT interval prolongation have also been observed (EMA, 2024).

However, evidence on the use of ivabradine off-label in the pediatric population is limited and primarily based on case reports or series, particularly in patients younger than 6 months. This poses a significant challenge, as higher prevalence, morbidity, and mortality rates have been reported in this age group.

In this context, the authors find it relevant to share our experience with ivabradine use in patients under 6 months of age to enhance the current evidence regarding its efficacy, safety, and dosing. This also aims to assist other healthcare professionals in its application. To date, this represents the largest published cohort of patients under 6 months receiving ivabradine treatment.

### 4.1 Ivabradine in HF

The only published clinical trial of ivabradine in pediatric patients was conducted by Bonnet et al. (2017); this study included pediatric patients aged 6 months and older with DCM and chronic symptomatic HF. Patients under 6 months of age were excluded due to potential inadequate tolerability with the concomitant administration of two antiarrhythmic drugs. Ivabradine was shown to significantly improve HR, cardiac function, LVEF, and patient quality of life, with an acceptable safety profile. The overall reduction in HR was 21%, and in the subgroup of patients aged six to 12 months, it was 25%. Patients exhibited an increase in LVEF of 11.4% at 6 months of treatment and 13.5% at 12 months. In our cohort of patients with symptomatic HF, a statistically significant reduction in HR was observed, along with an improvement in cardiac function, although the increase in LVEF was smaller. Bradycardia was reported in 11% of patients in the clinical trial, a value comparable to that observed in our cohort (n = 1, 14%).

Most patients in the trial received ivabradine in combination with other pharmacological therapies, including ACEIs, diuretics, beta-blockers, and digoxin. In the present study, all patients started ivabradine in conjunction with other treatments, and only in one case was it possible to discontinue these additional therapies by the end of the ivabradine treatment.

Due to the high interindividual variability observed in treatment response, Bonnet et al. (2017) emphasized the importance of appropriate dose titration. In the subgroup of patients aged six to 12 months (n = 10), an initial dose of 0.02 mg/kg/day every 12 h was established, with dose escalation up to a maximum dose of 0.2 mg/kg/day. In our cohort, the median initial dose used (0.06 mg/kg/day every 12 h) was higher than that reported in the trial, while the maintenance dose was similar (0.2 mg/kg/day every 12 h).

In the literature review conducted, no published cases or series of cases regarding the use of ivabradine in patients under 6 months of age with a diagnosis of HF were found. Therefore, this study is the first to report on the use of ivabradine in this population, making it essential for the authors to share the obtained results. The study demonstrates favorable outcomes regarding the efficacy and safety of ivabradine in this cohort. Additionally, the dosing guidelines used may provide valuable guidance for other healthcare professionals in its application.

### 4.2 Ivabradine in SVT

The use of ivabradine in pediatric patients with SVT is not well-defined, and current experience is based on case reports and series. The observed results are promising, especially in patients refractory to conventional antiarrhythmics, allowing for complete reversion to sinus rhythm with an acceptable safety profile (Younis et al., 2021; Tolani et al., 2024). Most cases involve patients aged 1 year and older, with evidence for patients under 6 months (Michel et al., 2020; Kumar et al., 2017; Bohora et al., 2011; Janson et al., 2019; Gul et al., 2023; Dasgupta and Johnsrude, 2023), being even more limited. Table 4 summarizes publications that include patients under 6 months of age who have received ivabradine treatment for various types of SVT.

**TABLE 4:** Publications including patients under 6 months of age who have received ivabradine treatment for a diagnosis of any type of SVT.

References and type of publication	Indication	n	Age	Administration regimen	Ivabradine dose (mg/kg/day every 12 h)		Results
					Initial	Maintenance	
Dieks et al. (2016) Prospective study	Resistant congenital JET	5	10 d - 3.5 y (median: 8 m)	Adjuvant therapy	0.05 - 0.1	0.22	- Resolution of JET in all patients (restoration to sinus rhythm in four patients) - No adverse effects
Ergul et al. (2018) Case series	Congenital JET and secondary multidrug-resistant cardiomyopathy	3	52 d, 2 m, 10 m	Adjuvant therapy	0.1	2 patients: 0.1 1 patient: 0.2	- Response within 24 h of initiation - Complete reversal to sinus rhythm in two patients and partial reversal in one - No adverse effects
Memon et al. (2022) Clinical case	Resistant congenital JET	1	14 d	Adjuvant therapy	0.1	0.1	- Rapid resolution of tachycardia following initiation - No adverse effects
Asfour et al. (2021) Clinical case	Resistant congenital JET	1	26 d	Adjuvant therapy	0.1	0.08	- Reversion to sinus rhythm after 3 days of treatment - Bradycardia requiring dose adjustment - After 6 months of follow-up, adequate control of HR with no adverse effects
Ríos et al. (2021) Clinical case	Resistant congenital JET	1	12 d	Adjuvant therapy	0.1	0.1	- Reversion to sinus rhythm and discontinuation of one of the two antiarrhythmic medications - No adverse effects
Devaprasath et al. (2022) Clinical case	Resistant congenital JET	1	Neonate	Monotherapy	0.2	0.2	- Reversion to sinus rhythm within 2 h of initiation - No adverse effects
Khan et al. (2020) Prospective study	Resistant postoperative JET	7	1 patient: 5 m 2 patients: 6 m	Adjuvant therapy	0.1	0.1	- Time between diagnosis and initiation of treatment: 5–8 h - Reversion to sinus rhythm within 1–15 h of initiation - Duration of treatment: 2 doses - No adverse effects
Krishna et al. (2019) Retrospective study	Resistant postoperative JET	8	1 patient: 3 d 1 patient: 1 m 2 patients: 2 m	Monotherapy	0.1	0.1	- Reversion to sinus rhythm within 3–16 h after initiation - Duration of treatment: 3–4 doses - Adequate HR control and reversion to sinus rhythm - No adverse effects
López Fernández et al. (2021) Case series	Resistant postoperative JET	3	2.5 m, 5 m, 5.6 m	Adjuvant therapy	0.2	0.2	- Reversion to sinus rhythm within 48–96 h after initiation - Duration of treatment: 3–4 d - No adverse effects
Younis et al. (2021) Retrospective study	SVT *79 patients with SVT were included, with ivabradine administered to 3 of them for resistant SVT. Only one patient was <6 months of age	1	19 d	Adjuvant therapy	0.05	0.075	- Reversion to sinus rhythm after dose escalation (0.075 mg/kg/day every 12 h) on day 2 - No adverse effects - Ivabradine appears to be a safe and well-tolerated medication that can induce adequate suppression of SVT, achieve complete reversion to sinus rhythm, and effectively improve left ventricular function
Xu et al. (2023) Prospective study	Resistant FAT	12	5 m −15 y (a 5-month-old patient)	Monotherapy	0.2	0.4	- Reversion to sinus rhythm after 48 h of treatment in 6 patients (including the 5-month-old patient) - No adverse effects
Tolani et al. (2024) Retrospective study	TAF in patients with CHD	15	7 m (1–18 m) (3 patients <3 m)	Adjuvant therapy 11 patients Monotherapy 4 patients	0.05	0.07 (0.04–0.16)	- First-line treatment in 5 patients - Reversion to sinus rhythm in 12 patients within the first 24 h after initiation - Bradycardia in 7 patients
Tasci and Karadeniz (2023) Clinical case	Resistant FAT	1	15 d	Adjuvant therapy	0.1	0.1	- Reversion to sinus rhythm and discontinuation of 2 out of 4 antiarrhythmic medications after one dose - No adverse effects
Cohen et al. (2020) Clinical case	MAT	1	5 m	Adjuvant therapy	0.05	0.05	- Duration of treatment: 2 doses - Stabilization and reversion to a single EAT, enabling subsequent ablation and reversion to sinus rhythm - No adverse effects
Karmegaraj et al. (2021) Clinical cases	Resistant EAT	2	60 d	Adjuvant therapy	0.3	0.3	- Reversion to sinus rhythm within 12 h of initiation - HR control and hemodynamic stability after one dose - No adverse effects
	EAT		30 d	Adjuvant therapy	0.3	0.3	- Early initiation after diagnosis - Reversion to sinus rhythm within 4 h of initiation - No adverse effects
Penslar and Udupa (2023) Clinical case	Resistant EAT	1	42 d	Monotherapy	0.05	0.3	- Reversion to sinus rhythm - Duration: 1 year, currently in the process of discontinuation - No adverse effects

CHD: congenital heart disease; d: days; EAT: ectopic atrial tachycardia; HR: heart rate; FAT: focal atrial tachycardia; JET: junctional ectopic tachycardia; m: months; MAT: multifocal atrial tachycardia; SVT: supraventricular tachycardia; y: years.

JET is considered an uncommon SVT, which can be congenital (congenital JET) or post-surgical (post-surgical JET), with the latter being more common (Younis et al., 2021; Kumar et al., 2019; Dieks et al., 2016). Despite its lower incidence, congenital JET is associated with severe cardiovascular complications and high morbidity and mortality, particularly in patients under 6 months of age with elevated heart rates, who have a worse prognosis (Younis et al., 2021; Dieks et al., 2016). To achieve adequate HR control, the administration of at least two antiarrhythmic drugs is typically required, with amiodarone being the treatment of choice (Dieks et al., 2016). However, the lack of efficacy of conventional treatments has led to the use of off-label medications such as ivabradine, which, although its experience is limited, has shown favorable results (Younis et al., 2021). Current studies conclude that ivabradine is an effective therapeutic option with a good safety profile for managing resistant congenital JET, providing adequate HR control and complete reversion to sinus rhythm (Dieks et al., 2016; Ergul et al., 2018; Ríos et al., 2021; Devaprasath et al., 2022; López Fernández et al., 2021). Additionally, it may reduce the need for combinations of more than two antiarrhythmic drugs and invasive treatments (Ríos et al., 2021). While ivabradine is often administered in combination with other antiarrhythmic agents, it has also demonstrated adequate efficacy as monotherapy (Devaprasath et al., 2022). Some authors suggest that early initiation of treatment following diagnosis may be related to the positive response (Dieks et al., 2016).

Post-surgical JET is the most common post-surgical arrhythmia that causes hemodynamic instability, and it is associated with significant morbidity and mortality (Kumar et al., 2019). One of the risk factors for its development is young age (Kumar et al., 2019). Its treatment typically involves intravenous pharmacotherapy with agents such as amiodarone and flecainide (Kumar et al., 2019). Similar to congenital JET, ivabradine has shown promising results in terms of efficacy and safety for the treatment of post-surgical JET, both as an adjunctive therapy and as monotherapy, with a notable rapid onset of action (Kumar et al., 2019; Khan et al., 2020; Krishna et al., 2019; López Fernández et al., 2021). In this case, studies also recommend early initiation of treatment (Michel et al., 2020; López Fernández et al., 2021).

In cases of FAT, MAT, or EAT that are refractory to conventional treatments, off-label use of ivabradine has also yielded results similar to those observed with JET; so, ivabradine may be an effective and safe therapeutic option, either in conjunction with other antiarrhythmic agents or as monotherapy. Additionally, there is a noted association between early initiation of ivabradine and a more rapid reversion to sinus rhythm (Younis et al., 2021; Tolani et al., 2024; Karmegaraj et al., 2021). The only adverse effect reported in the studies has been bradycardia, which underscores the importance of close monitoring (Tolani et al., 2024).

Consistent with the reviewed publications, this study observed a statistically significant reduction in HR among patients receiving ivabradine for SVT. Additionally, ivabradine could be withdrawn in four patients (57%) following reversion to sinus rhythm. The median starting dose of ivabradine was 0.1 (0.1 - 0.12) mg/kg/day every 12 h, with a median maintenance dose of 0.24 (0.12 - 0.32) mg/kg/day every 12 h, results comparable to those reported in the literature. The median time from diagnosis to initiation of ivabradine was 3 days, which can be considered an early initiation of treatment, a relevant factor as it may influence the response. The only adverse effect observed in this study was bradycardia in two patients, an event previously reported by other authors, but it did not lead to discontinuation of treatment in any patient. Although the overall median number of concomitant treatments was not reduced, it was possible to discontinue some of the initial therapies in five patients (71%).

### 4.3 Study limitations

The primary limitations and biases of the study arise from its retrospective nature, such as the lack of data in some cases. However, despite the limited number of patients, this is currently the first published study that includes patients under 6 months of age with symptomatic HF treated with ivabradine and represents the largest cohort of patients under 6 months receiving ivabradine for the diagnosis of SVT.

## 5 Conclusion

In conclusion, in patients under 6 months of age with symptomatic HF or SVT, ivabradine improved clinical parameters with a satisfactory safety profile and proved to be an effective and safe therapeutic alternative for this patient population.

## Data Availability

The raw data supporting the conclusions of this article will be made available by the authors, without undue reservation.

## Glossary

ACEIs: Angiotensin-converting enzyme inhibitors
AV: Atrioventricular
bpm: Beats per minute
CHD: Congenital heart diseases
d: Days
DCM: Dilated cardiomyopathy
EAT: Ectopic atrial tachycardia
EMA: European Medicines Agency
FAT: Focal atrial tachycardia
FDA: Food and drug administration
GW: Gestational weeks
HF: Heart failure
HR: Heart rate
JET: Junctional ectopic tachycardia
LV: Left ventricular
LVEF: Left ventricular ejection fraction
M: Man
MAT: Multifocal atrial tachycardia
m: Months
NA: Not available
NG: Nasogastric
NIOSH: National Institute for Occupational Safety and Health
OR: Oral
RV: Right ventricular
SD: Standard deviation
SVT: Supraventricular tachycardias
W: Woman
y: Years
